# Acute liver failure related to hepatitis B (a four-case series)

**DOI:** 10.1186/1471-2334-13-S1-P100

**Published:** 2013-12-16

**Authors:** Andrei Rogoz, Cleo Roşculeț, Cătălin Apostolescu, Monica Ivan, Marius Radu, Aida Răşcanu, Alina Frangulea, Monica Ungureanu, Mihaela Tudor, Bogdan Pavel, Doina Iovănescu

**Affiliations:** 1 National Institute for Infectious Diseases “Prof. Dr. Matei Balş”, Bucharest, Romania

## Background

Acute liver failure related to hepatitis B infection may occur after acute HBV infection or during an exacerbation (flare) of a chronic HBV infection. The condition is associated with a very high morbidity and mortality rate.

## Case report

We present the diagnosis and management difficulties of a four-case series admitted to our clinic during the last year, with severe acute liver failure, with encephalopathy that required medical management, intensive care and organ support, including artificial extracorporeal liver support.

## Conclusion

In the presence of acute liver failure and serological evidence of hepatitis B viral infection, differentiating between acute infection and exacerbation (flare) of chronic infections remains a challenge. Mortality rates remain high inspite of recent medical advance.

